# Structure, Martensitic Transformation, and Damping Properties of Functionally Graded NiTi Shape Memory Alloys Fabricated by Laser Powder Bed Fusion

**DOI:** 10.3390/ma15145073

**Published:** 2022-07-21

**Authors:** Hao Jiang, Rui Xi, Xiaoqiang Li, Sergey Kustov, Jan Van Humbeeck, Xiebin Wang

**Affiliations:** 1 Key Laboratory for Liquid-Solid Structural Evolution and Processing of Materials, Ministry of Education, Shandong University, Jingshi Road 17923, Jinan 250061, China; hao.jiang2020@hotmail.com (H.J.); rui.xi2020@hotmail.com (R.X.); lixiaoqiang3314@163.com (X.L.); 2 Departament de Física, Universitat de les Illes Balears, Cra Valldemossa km 7.5, E07122 Palma de Mallorca, Spain; sergey.kustov@uib.es; 3 Department of Materials Engineering, University of Leuven (KU Leuven), Kasteelpark Arenberg 44 Bus 2450, B3001 Heverlee, Belgium; jan.vanhumbeeck@kuleuven.be

**Keywords:** shape memory alloy, NiTi, additive manufacturing, laser powder bed fusion, functionally graded materials, damping

## Abstract

Besides the unique shape memory effect and superelasticity, NiTi alloys also show excellent damping properties. However, the high damping effect is highly temperature-dependent, and only exists during cooling or heating over the temperature range where martensitic transformation occurs. As a result, expanding the temperature range of martensite transformation is an effective approach to widen the working temperature window with high damping performance. In this work, layer-structured functionally graded NiTi alloys were produced by laser powder bed fusion (L-PBF) alternating two or three sets of process parameters. The transformation behavior shows that austenite transforms gradually into martensite over a wide temperature range during cooling, and multiple transformation peaks are observed. A microstructure composed of alternating layers of B2/B19′ phases is obtained at room temperature. The functionally graded sample shows high damping performance over a wide temperature range of up to 70 K, which originates from the gradual formation of the martensite phase during cooling. This work proves the potential of L-PBF to create NiTi alloys with high damping properties over a wide temperature range for damping applications.

## 1. Introduction

Besides the unique shape memory effect (SME) and superelasticity (SE), NiTi shape memory alloys (SMAs) are also attracting increasing interest due to their excellent damping properties [1,2,3,4,5]. The damping, or internal friction (IF), reflects the ability of materials to dissipate the energy of mechanical oscillations. The damping of NiTi alloys is mainly contributed by the following two parts: (i) martensitic transformation, and (ii) high mobility of martensite twin boundaries [6,7]. The former part gives rise to the transitory and the phase transition component. The contribution of martensitic transformation exists only during cooling or heating in a certain temperature range, which is related highly to the martensite transformation interval [8,9], e.g., the temperature range between the martensite transformation start (*M_s_*) and finish (*M_f_*) temperatures during cooling. As a result, expanding the transformation interval is essential to obtain high damping over a wide temperature range.

Developing functionally graded NiTi alloys is a promising approach to obtain high damping properties through expanding the transformation interval [10,11,12]. It is well-known that the martensite transformation temperature (MTT) of near equiatomic NiTi alloys depends highly on the Ni content of the matrix, and the MTTs decrease remarkably with the increase of Ni concentration on the Ni-rich side [1,13,14,15]. As a result, the functionally graded NiTi alloys fabricated by gradually changing the Ni concentration have been addressed [10,11,16,17,18,19,20,21,22,23]. Since it is quite challenging to tailor the Ni content with an accuracy of 0.1 at. % through conventional metallurgical methods (e.g., casting), diffusion annealing has been applied to produce compositionally graded NiTi alloys. Hu et al. [10] obtained a high damping plateau (larger than 0.04) over a wide temperature window (>110 K) in a compositionally graded NiTi plate prepared by electroplating of Ni coating on NiTi substrate, followed by diffusion annealing. Meng et al. [11] also prepared the compositionally graded NiTi plates by diffusion annealing using NiTi and pure Ni plates, which show a wide transformation interval of >70 K. Selective vaporization of Ni element by laser processing has also been applied to produce the compositionally graded NiTi alloys [24,25]. However, the application of the diffusion annealing, and the laser processing techniques are limited largely by the size and shape of NiTi samples. They are mainly applicable for thin wires and plates.

Recently, the fabrication of NiTi SMAs by additive manufacturing (AM) has been frequently addressed [26,27,28,29,30,31], due to its ability to fabricate geometrically complex NiTi structures, which are quite challenging to fabricate via conventional approaches (e.g., casting, machining). Among the AM technologies, laser powder bed fusion (L-PBF) has attracted much attention [32,33,34,35,36,37] due to the fact that L-PBF fabricated parts normally have a higher dimensional accuracy and better surface finish. Moreover, L-PBF also shows a great potential to act as a metallurgical method to modify the microstructure and phase transformation temperatures of NiTi alloys. For instance, in our previous work, it was found that the MTTs of L-PBF fabricated NiTi alloys increase monotonously with the increase of laser power (*P*), the decrease of scanning speed (*v*) or hatch spacing (*h*), mainly due to the increased Ni-loss by evaporation under high energy density [38,39,40,41]. This demonstrates that LPBF is a feasible method to modify the Ni concentration and, thus, the transformation temperature of NiTi alloys. The layer-by-layer building permits the L-PBF method of fabricating NiTi specimens with different MTTs at different locations; thus producing the functionally graded NiTi alloys [42,43].

In our previous work [42], a layer-structured functionally graded NiTi alloy with high damping performance was produced by alternatively changing the L-PBF process parameters. However, the influence of the selected L-PBF process parameters and layer thickness on the microstructure, phase transformation behavior, and damping properties has not been well studied yet. In order to explore the potential of this method to develop functionally graded NiTi alloys, the abovementioned issues are systematically studied in this work. This work suggests that the L-PBF process parameters and the layer thickness must be carefully selected and optimized in order to develop functionally graded NiTi alloys with improved damping performance. The high damping properties over a wide temperature window will promote largely the applications of NiTi alloys.

## 2. Materials and Methods

**Figure 1:**
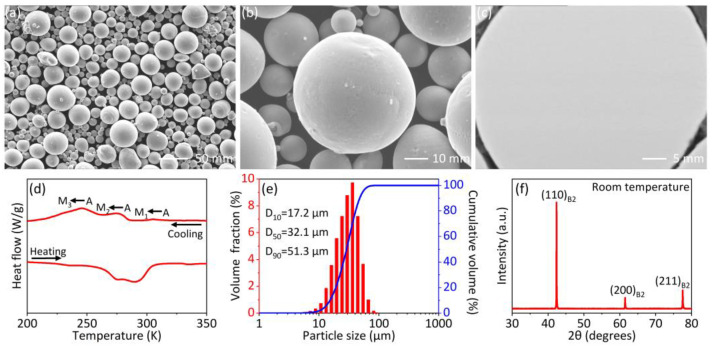
(**a**) and (**b**) SEM images of NiTi powders, (**c**) SEM image of the cross-section of a NiTi particle in backscattered mode; (**d**) phase transformation behavior, (**e**) particle size distribution, and (**f**) X-ray diffraction pattern of the NiTi powders.

Spherical NiTi alloy powders supplied by Minatech Co., Ltd. (Shenzhen, China) were used in this study. The composition of the powders is shown in Table 1. The scanning electron microscopy (SEM) images of the powders are shown in Figure 1a–c. The majority of the powder particles are spherical, and only a few irregular and satellite particles are observed. As shown in Figure 1c, the backscattered image (BSE) of the cross-section of a NiTi particle demonstrates no obvious secondary phase exists. Figure 1d shows that three phase transformation peaks are observed during cooling, mainly due to the composition inhomogeneity of the powders. Figure 1e shows a particle size ranging 3.9 to 90.8 µm (*D*_50_ of 32.1 µm), measured by a Bettersize 2600 particle size analyzer (Bettersize, Dandong, China). Particles are indexed as B2 phase at ambient temperature (Figure 1f).

The NiTi parts were fabricated using a 3D Systems ProX DMP 320 L-PBF system (3D Systems, Rock Hill, CA, USA) with maximum power of 500 W and beam size of 60 μm. All samples were fabricated under high-purity argon atmosphere (99.999%) with an oxygen content of <25 ppm. A rotated stripe scanning strategy was employed. The stripe width was 5 mm, and the rotation between successive layers is 57°.

**Figure 2:**
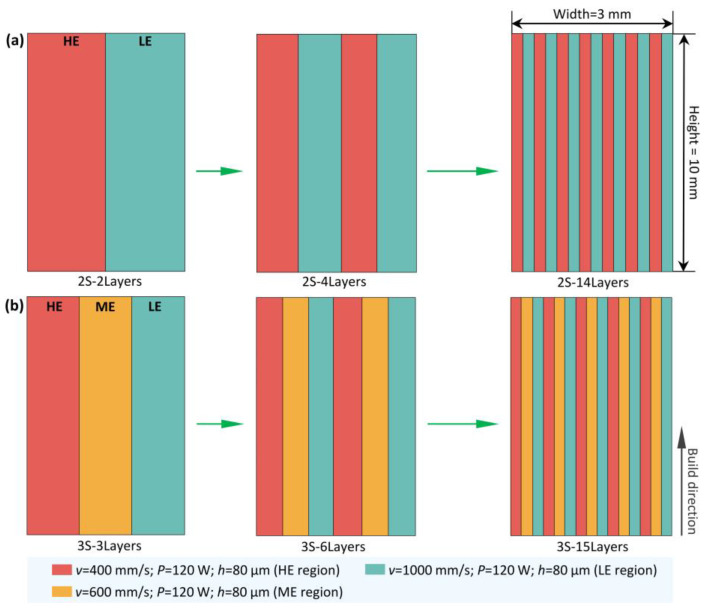
Schematic drawings of the layer-structured samples fabricated using (**a**) 2 sets of L-PBF process parameters and (**b**) 3 sets of L-PBF process parameters with different total number of layers. The LE, HE, and ME regions represent the layers fabricated with low energy density (*v* = 1000 mm/s), high energy density (*v* = 400 mm/s) and medium energy density (*v* = 600 mm/s), respectively. For all samples, the laser power and hatch spacing were fixed at 120 W and 80 µm, respectively.

In this work, the functionally graded NiTi samples, featured with layer-structure, were fabricated by periodically changing two sets (named as 2S samples) or three sets (named as 3S samples) of L-PBF process parameters. Figure 2 shows schematically the construction of layered samples. As shown in Figure 2a, two scanning speeds (*v* = 400 mm/s and *v* = 1000 mm/s) were selected to fabricate the 2S samples (laser power was kept at 120 W and hatch spacing was kept at 80 µm). The HE region and LE regions represent the layers fabricated with high energy density (i.e., *v* = 400 mm/s) and low energy density (i.e., *v* = 1000 mm/s), respectively. Figure 2b illustrates the strategy for producing the 3S samples. Scanning speeds of 400 (HE region), 600 (medium energy density region, ME region), and 1000 mm/s (LE region) were selected. The functionally graded NiTi samples have the same size of 60 × 10 × 3 mm^3^. The layer width depends on the total number of layers, since all the samples have the same width of 3 mm. Table 2 gives the detailed information of the L-PBF process parameters for producing the functionally graded NiTi samples. The samples fabricated with a single set of L-PBF process parameters (i.e., V1, V2 and V3 in Table 2) were used as a reference.

The microstructure of L-PBF fabricated NiTi samples was studied using a JSM-7800 scanning electron microscope (SEM, JEOL, Tokyo, Japan). Phase composition at room temperature was determined using a DMAX-2500PC X-ray Diffractometer (XRD, Rigaku, Japan) with Cu Kα radiation under a scanning speed of 10°/min. Electron backscatter diffraction (EBSD) analysis was conducted to study the crystallographic texture using a JSM-7800 microscope (JEOL, Tokyo, Japan) equipped with a NordlysMax3 system (Oxford Instruments, Abingdon, UK). The surface for EBSD observations was electropolished using a solution of 21 vol.% HClO_4_ and 79 vol.% CH_3_COOH. All the microstructure observations are taken from the surface perpendicular to the build direction. A DSC 204 differential scanning calorimeter (DSC, Netzsch, Selb, Germany) was used to study the martensitic transformation behavior under a heating/cooling rate of 10 K/min. The damping properties between 183 and 393 K were registered using a DMA 850 dynamic mechanical analyzer (DMA, TA Instruments, New Castel, DE, USA) under a heating/cooling rate of 5 K/min. Dual-cantilever mode was employed to study the damping properties, and the strain amplitude and frequency were fixed at 3 × 10^−3^ and 1 Hz, respectively.

## 3. Results and Discussions

### 3.1. Microstructure

**Figure 3:**
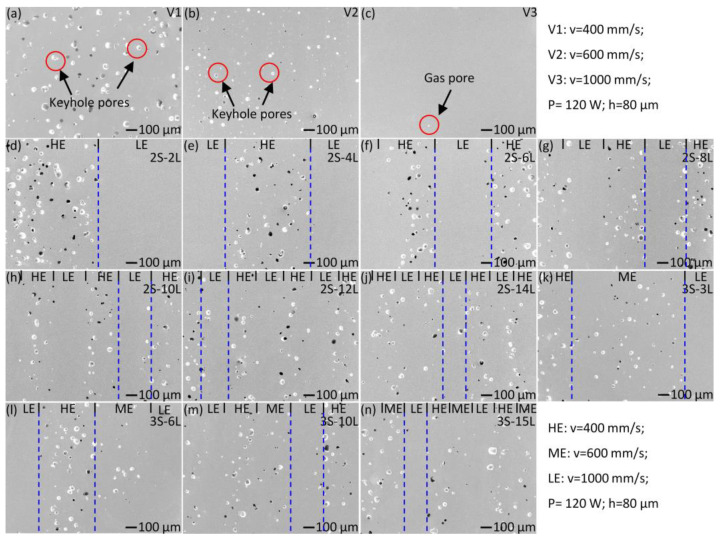
SEM secondary electron images, taken from the surfaces perpendicular to the build direction of the L-PBF fabricated NiTi samples with (**a**) *v* = 400 mm/s (sample V1 in Table 2), (**b**) 600 mm/s (sample V2), and (**c**) 1000 mm/s (sample V3); (**d**–**j**) are the layer-structured samples fabricated using 2 sets of L-PBF parameters with different total number of layers: (**d**) 2 layers (sample 2S–2L), (**e**) 4 layers (sample 2S-4L), (**f**) 6 layers (sample 2S-6L), (**g**) 8 layers (sample 2S–8L), (**h**) 10 layers (sample 2S-10L), (**i**) 12 layers (sample 2S-12L), and (**f**) 14 layers (sample 2S–14L); (**k**–**n**) are the layer-structured samples fabricated using 3 sets of L-PBF parameters with different total numbers of layers: (**k**) 3 layers (sample 3S-3L in Table 2), (**l**) 6 layers (sample 3S-6L), (**m**) 10 layers (sample 3S–10L), and (**n**) 15 layers (sample 3S–15L). The LE, HE, and ME regions represent the layers fabricated with low energy density (*v* = 1000 mm/s), high energy density (*v* = 400 mm/s), and medium energy density (*v* = 600 mm/s), respectively. The laser power of 120 W and hatch spacing of 80 µm are fixed.

The microstructure of the sample fabricated under the scanning speeds of 400, 600, and 1000 mm/s (laser power and hatch spacing are fixed at 120 W and 80 µm, respectively) is shown in Figure 3a–c, respectively. A large number of spherical pores are observed in the sample fabricated with a low scanning speed (e.g., *v* = 400 mm/s, sample V1). The large pores are considered keyhole porosities, which have been reported frequently in L-PBF fabricated metallic materials [44,45,46]. The keyhole pore is filled with metal vapor and shielding gas because the recoil pressure of the metal vapor pushes the surface toward the bulk metal. The large keyhole pores can be minimized largely by reducing the input energy density. As indicated in Figure 3b, the pores are reduced obviously by increasing the laser scanning speed to 600 mm/s (sample V2). No keyhole pores are observed in the sample fabricated with *v* = 1000 mm/s (i.e., sample V3, Figure 3c). Only a few gas pores are observed in sample V3, which originates from entrapping the gas from the protection of Ar gas [47,48].

Figure 3d–j show the microstructure of the samples fabricated with two sets of L-PBF parameters, which change periodically. The LE and HE regions are manufactured with *v* = 400 and 1000 mm/s, respectively, while the laser power is fixed at 120 W and hatch spacing is fixed at 80 µm. Figure 3d shows that a clear boundary between the HE and LE regions could be identified, where plenty of keyhole pores exist in the HE region. The width of LE and HE layers decreases with the increase of the total number of layers, since the overall width of the sample is fixed as 3 mm. Meanwhile, the boundary between the LE and HE regions gradually blurs with the increase of total numbers of layers. As shown in Figure 3j, large keyhole pores are observed in the entire surface of the 2S–14L sample, which has a pre-set layer width of 200 µm. However, the parallel aligned large pores are indicative of the layer-structures.

Figure 3k–n give the microstructure of the samples fabricated by periodically changing the three sets of L-PBF parameters, i.e., *v* = 400 (HE region), 600 (ME region), and 1000 mm/s (LE region). Large keyhole pores are observed in both the HE and ME regions, while the size and density of the keyhole pores are lower in the ME region than in the HE region. The width of each layer decreases with the increase of the total number of layers. Figure 3m shows that the parallel aligned large keyhole pores (HE region), small keyhole pores (ME region), and nearly pore-free region (LE region) can still be identified for the 3S–10L sample, which has a pre-set layer width of 300 µm. The boundaries between the HE and ME regions are not distinguishable for the 3S-15L sample (Figure 3n), but the thin nearly pore-free region (LE region) can still be observed.

The above results indicate that the functionally graded NiTi alloys featured with the layered microstructure could be obtained by periodically changing the L-PBF process parameters. However, the HE region likely has a wider influence area than the pre-set width, leading to the decrease of the LE region with the decrease of layer width. As a consequence, the difference between the layers vanishes gradually with the decrease of layer width, resulting in a more homogeneous microstructure (Figure 3j,n).

**Figure 4:**
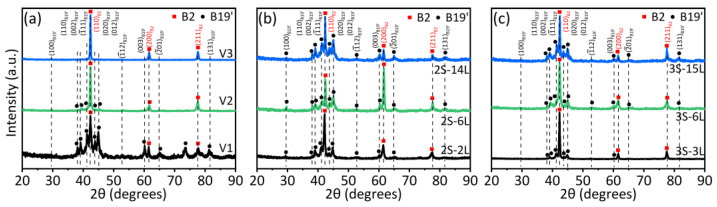
XRD patterns of (**a**) the samples manufactured with *v* = 400 mm/s (sample V1), 600 mm/s (sample V2), and 1000 mm/s (sample V3); (**b**) the layer-structured samples manufactured using 2 sets of L-PBF parameters with total number of 2 layers (sample 2S–2L), 6 layers (sample 2S–6L), and 14 layers (sample 2S–14L); (**c**) the layer-structured samples manufactured using 3 sets of L-PBF parameters with total number of 3 layers (sample 3S–3L), 6 layers (sample 3S–6L), and 15 layers (sample 3S–15L), respectively. For all samples, XRD patterns are collected at ambient temperature; the laser power of 120 W and hatch spacing of 80 µm are fixed.

Figure 4 gives the phase composition of the functionally graded NiTi alloys, as revealed by XRD at room temperature. Figure 4a indicates the sample produced with a low scanning speed of 400 mm/s (sample V1) contains mainly B19′ martensite at room temperature, since the B2 reflections are very weak. The volume fraction of the B19′ phase decreases with the increase of scanning speed, as suggested by the decrease of B19′ reflections. Only the B2 austenite is detected in the sample produced with v = 1000 mm/s (sample V3). The above results indicate that the MTTs gradually decrease with the increase of scanning speed and, thus, the increase of B2 phase at ambient temperature.

Figure 4b,c show the XRD patterns of the functionally graded samples fabricated with two and three sets of L-PBF process parameters, respectively. All the samples show dual-phase microstructure, since both B2 and B19′ reflections are detected. According to Figure 4a, the LE region is in the austenite phase, while the HE and ME regions are mainly in martensite at ambient temperature. Interestingly, the intensities of B19′ reflections increase with the increase of the total number of layers, suggesting an increase of the martensite phase at room temperature. This suggests that the overall MTTs of the functionally graded samples increase with the increase of the total number of layers, although the volume fraction of the LE regions is pre-set as the same in the 2S or 3S samples.

**Figure 5:**
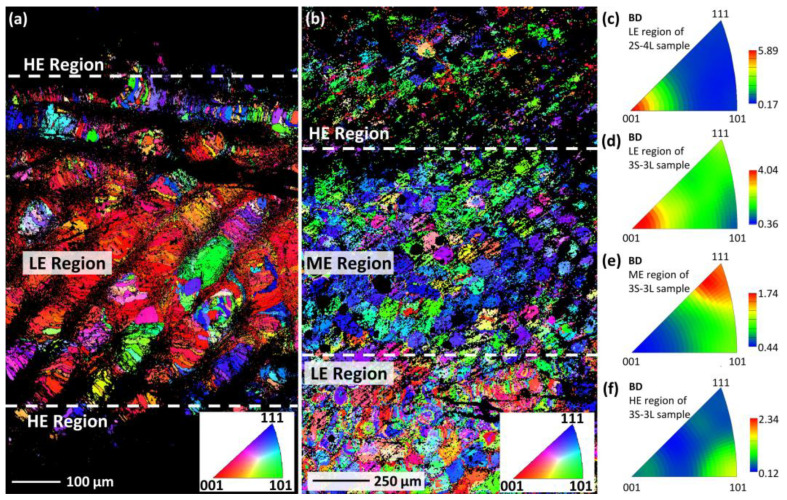
EBSD orientation maps, taken from the surface perpendicular to the build direction: (**a**) the layer-structured samples fabricated using 2 sets of L-PBF parameters with a total number of 4 layers (sample 2S–4L); (**b**) the layer-structured samples fabricated using 3 sets of L-PBF parameters with a total number of 3 layers (sample 3S–3L). Inverse pole figures show the texture with the reference to BD for the regions: (**c**) the LE region of the sample 2S–4L; (**d**) the LE region, (**e**) the ME region, (**f**) the HE region of the sample 3S-3L. BD represents the build direction. The LE, HE, and ME regions represent the layers fabricated with low energy density (*v* = 1000 mm/s), high energy density (*v* = 400 mm/s), and medium energy density (*v* = 600 mm/s), respectively. For all samples, the laser power is fixed at 120 W and hatch spacing is fixed at 80 µm.

Figure 5a shows the microstructure of the 2S-4L sample, as characterized by EBSD at room temperature. The black area is considered the B19′ martensite phase, since the martensite plates are very fine (<100 nm [1]), which makes it very difficult for the EBSD instrument to get clear Kikuchi patterns. It is clear that the alternative B19′/B2 layer-structured microstructure at room temperature is developed. According to the XRD results, the B19′ phase layer (i.e., the black area in Figure 5a) and the B2 phase layer correspond to the HE region and LE regions, respectively. A strong <100>_B2_//BD fiber texture is revealed in the LE region (Figure 5c), which has also been reported previously [35,49,50,51,52]. The development of the strong texture is mainly due to the epitaxial grain growth along the maximum temperature gradient, which is normally parallel to the BD [53,54,55]. Since the easy growth direction of the B2 ordered NiTi is <100>, the strong <100>_B2_//BD texture is developed.

Figure 5b shows the EBSD map of the 3S–3L sample, which is fabricated by changing alternately the scanning speeds of 400 (HE region), 600 (ME region), and 1000 mm/s (LE region). The layered microstructure is also revealed, since the volume fraction of B2 phase decreased with increasing scanning speed, as indicated by the increase of the un-indexed area (i.e., the black area in Figure 5b). The inverse pole figures of LE, ME, and HE regions are shown in Figure 5d–f, respectively. Interestingly, different textures are detected in the different regions. The type of texture in the LE, ME, and HE regions are identified as <100>_B2_//BD, <111>_B2_//BD, and <110>_B2_//BD, respectively. This suggests that the preferential growth direction of NiTi alloy is influenced largely by the scanning speed, since the LE, ME, and HE regions are fabricated under the same conditions, except for the laser scanning speed. The reason for this phenomenon is not well understood at present, and further studies are highly required.

### 3.2. Phase Transformation Behavior

**Figure 6:**
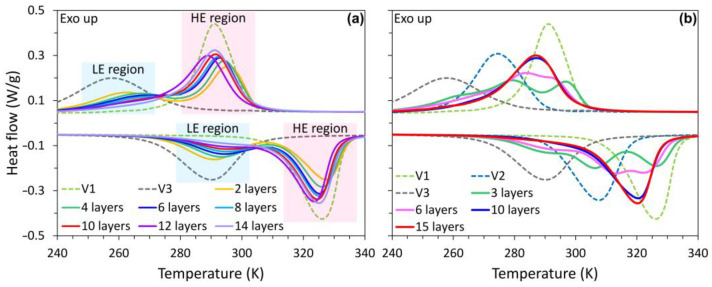
DSC scans showing the phase transformation behavior of: (**a**) the L-PBF fabricated NiTi samples with a *v* = 400 (sample V1) and 1000 mm/s (sample V3), and the layer-structured samples fabricated using 2 sets of L-PBF parameters (*v* = 400, 1000 mm/s) with a different total number of layers; (**b**) the layer-structured samples fabricated using 3 sets of L-PBF parameters (*v* = 400, 600, and 1000 mm/s) with a different total number of layers. For all samples, the laser power is fixed at 120 W and the hatch spacing is fixed at 80 µm.

Figure 6a shows the phase transformation characteristics of the functionally graded NiTi alloys fabricated by periodically changing the scanning speeds of 400 and 1000 mm/s (the laser power is fixed at 120 W and hatch spacing is fixed at 80 µm). The samples V1 and V3, fabricated with scanning speeds of 400 mm/s and 1000mm/s, respectively, are also presented as references. Both the V1 and V3 samples exhibit a typical one-stage martensitic phase transformation during either cooling or heating, which relates to the B2↔B19′ transformation. The samples V1 and V3 show a martensite transformation peak temperature (*M_p_*) at 291 K and 258 K, respectively. The MTTs decreased with increasing scanning speeds due to the reduced Ni-loss by evaporation under low energy density [56].

Two transformation peaks during both cooling and heating are observed in the functionally graded samples with the total number of layers <8 (Figure 6a). The peaks are identified as two pairs of A↔M transformation, due to the large transformation hysteresis [1]. Partial DSC tests were employed to identify the transformation peaks, as shown in Figure S1 in the Supplementary Materials. The A↔M transformation at the high temperature side is considered to occur in the HE region, which is fabricated with *v* = 400 mm/s. The A↔M transformation at the low temperature side takes place in the LE region. Figure 6a shows that the transformation peak of the LE region gradually decreases with the increase of the total number of layers, i.e., the decrease of layer width. Nearly only the transformation peak of the HE region could be observed for the samples with ≥10 layers. This also indicates that the HE region has a wider influence area than the pre-set width, resulting in the decrease of the LE region with the decrease of layer width, which is in accordance with the observations in Figure 3.

Figure 6b gives the DSC curves of the functionally graded samples produced with 3 sets of L-PBF process parameters, i.e., scanning speeds of 400 (HE region), 600 (ME region), and 1000 mm/s (LE region). Three pairs of A↔M transformation peaks are observed in the 3S–3L sample. The peaks with *M_p_* temperature at 269, 279, and 297 K correspond to the LE, ME, and HE regions, respectively. Similar to the results shown in Figure 6a, the transformation peaks gradually merge with the increase of the total number of layers. Only one transformation peak at 287 K (*M_p_* temperature) is observed for the samples 3S–10L and 3S–15L.

### 3.3. Damping Properties

The internal friction (IF) of materials undergoing first order structural transitions is conventionally interpreted in terms of three components [57,58]: (i) intrinsic damping of the martensite or austenite phases (*IF_int_*); (ii) transitory damping (*IF_tr_*) which exists only during phase transformation, *IF_tr_* depends strongly on external parameters, e.g., cooling/heating rate; (iii) phase transition component (*IF_pt_*), which exists also within the transformation range, but is associated with conventional reversible anelastic strain. Existing models for *IF_tr_* during thermoelastic martensitic transformation have been analyzed in Refs. [57,58]. As a first approximation, *IF_tr_* is proportional to the transformation rate ($\dot{T}$) and inversely proportional to the frequency (*f*) and oscillatory strain amplitude ($\varepsilon_{0}$):

(1) $$IF_{tr}=K\frac{\dot{T}}{\varepsilon_{0}f}$$

where *K* is a numerical factor. According to Equation (1), the *IF_tr_* disappears under isothermal conditions.

The phase transition *IF_pt_* component stems from the co-existence of the two phases and is ascribed to the direct contribution of the interphase boundaries and potential enhancement of the intrinsic damping during the transformation. In NiTi alloys, the *IF_pt_* term includes both linear (amplitude-independent) and non-linear (amplitude-dependent) terms. The non-linear component is substantial even for moderate strain amplitudes (e.g., $\varepsilon_{0}=10^{-4}$), and it increases nearly linearly with strain amplitude [59,60]. The transitory and phase transition components of NiTi systems, including Ni-Ti [61], Ni-Ti-Cu [62,63], and Ni-Ti-Fe [62], have been studied in detail by means of DMA. It was found that the *IF_tr_* part is higher, but in the same order of magnitude as the *IF_pt_* part at low frequency (e.g., 1 Hz) and slow cooling/heating rate (e.g., 3K [61]). Therefore, it is considered that both the *IF_tr_* and *IF_pt_* contribute to the high damping properties in this work. However, future studies are required to study the influence of oscillation frequency, strain amplitude, and cooling/heating rate on the damping properties of the layer-structured functionally graded NiTi alloys.

**Figure 7:**
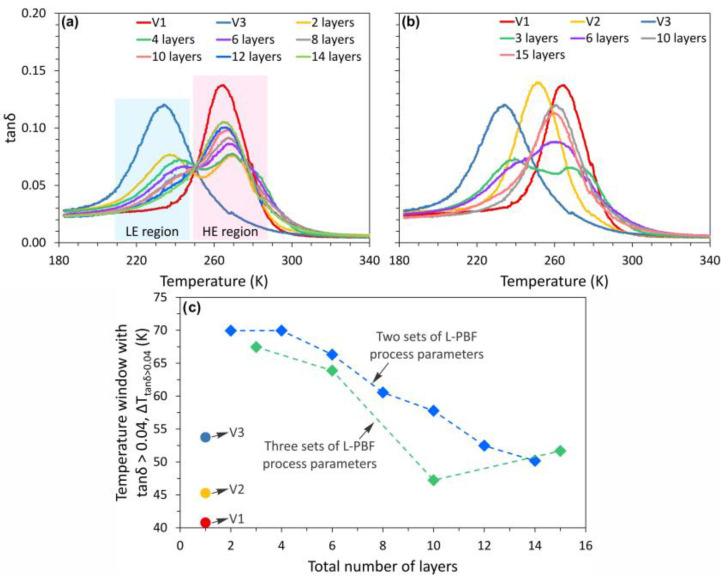
The internal friction (tan δ) of: (**a**) the L-PBF fabricated NiTi samples with *v* = 400 (sample V1) and 1000 mm/s (sample V3), and the layer-structured samples fabricated using 2 sets of L-PBF parameters (*v* = 400, 1000 mm/s) with a different total number of layers; (**b**) the L-PBF fabricated NiTi samples with *v* = 400 (sample V1), 600 (sample V2), and 1000 mm/s (sample V3), and the layer-structured samples fabricated using 3 sets of L-PBF parameters (*v* = 400, 600, and 1000 mm/s) with a different total number of layers. (**c**) The temperature window with tan δ > 0.04 (Δ*T*_tanδ__> 0.04_) as a function of the total number of layers. For all samples, the laser power is fixed at 120 W and hatch spacing is fixed at 80 µm.

Figure 7a shows the IF (expressed as tan δ) at 1 Hz during cooling for the functionally graded NiTi samples fabricated with two sets of L-PBF process parameters (see Table 2). The IF during heating process and the storage modulus during both the cooling and heating process are shown in Figures S2 and S3 in the Supplementary material. The data for the samples V1 and V3 is also presented for comparison. The V1 and V3 samples show high IF peaks at 265 and 235K, respectively. It is worth mentioning that the temperature of the DSC peaks and the IF peaks is different. For instance, the V1 sample has *M_p_* temperatures at 291 K, while the IF peak is observed at 265 K. The difference between the transformation temperatures as detected by DSC and DMA is typical [64] and attributed to the high thermal inertia of the DMA samples. Two IF peaks are observed for the samples with total number of layers ≤8. According to the DSC results in Figure 6a, the IF peak at the high temperature side occurs in the HE region, while the LE region gives the IF peak at the low temperature side. The two IF peaks gradually merge with the increase of the total number of layers to ≥10, in agreement with a more homogeneous microstructure obtained with the decrease of layer width.

The damping properties of the samples fabricated using 3 sets of L-PBF process parameters are shown in Figure 7b. Two IF peaks are observed in the 3S-3L sample, although three transformation peaks are detected by DSC (Figure 6b). Similar to the samples shown in Figure 7a, the peaks merge together with the decrease of layer width, and the samples with 10 and 15 layers show only one IF peak during cooling.

The working temperature window is important for applications of high damping materials. Figure 7c shows the temperature interval wherein tan δ > 0.04 (Δ*T*_tanδ__> 0.04_) for the functionally graded samples. The results for samples V1, V2, and V3 are also presented for comparison. The Δ*T*_tanδ__> 0.04_ of sample V1, V2, and V3 are 41, 45, and 54 K, respectively. The increase of Δ*T*_tanδ__> 0.04_ corresponds to the broadening of the transformation peaks with the increase of scanning speed (Figure 6). The Ni-loss caused by evaporation gradually increases with decreasing scanning speed. As a result, the sample fabricated with a high scanning speed (e.g., sample V3) has a higher Ni content than that fabricated with a low scanning speed (e.g., sample V1). It is suggested that nano-precipitates (or their nuclei) would form due to the cyclic heating process during L-PBF, which will broaden the transformation peaks.

Figure 7c shows that the layer-structured functionally graded samples possess much wider Δ*T*_tanδ__> 0.04_ than the sample produced with a single set of L-PBF process parameters. For instance, the 2S-2L and 3S-3L samples show Δ*T*_tanδ__> 0.04_ of 70 and 67 K, respectively. This indicates that the construction of the functionally graded microstructure by L-PBF is an efficient approach to expand the working temperature window of the NiTi alloys with high damping performance. However, Δ*T*_tanδ__> 0.04_ decreases gradually with the increase in the total number of layers. The 2S-14L and 3S-15L samples show Δ*T*_tanδ__> 0.04_ of 50 and 51 K, respectively, which are comparable to sample V2 (Δ*T*_tanδ__> 0.04_ = 45 K). The decrease of the working window for the sample with high number of layers is consistent with the homogenization of microstructure, which is revealed also by DSC. Therefore, we conclude that obtaining the widest working window for high damping L-PBF structures requires optimization of the parameters of the fabrication process.

## 4. Summary and Conclusions

In this work, layer-structured NiTi alloys were fabricated by periodically changing the selected L-PBF process parameters. The width of the layers was modified by changing the total number of layers, while keeping the overall width of the sample fixed. The evolution of the microstructure, transformation behavior, and damping properties with regard to the layer width were studied, and the main conclusions can be summarized as follows:

1. Layer-structured functionally graded NiTi alloys are produced by applying two or three alternating sets of L-PBF process parameters. Large keyhole poles are observed in the layers fabricated with high energy density (i.e., HE region), and the layers fabricated with low energy density are nearly defect-free.
2. The difference between the layers vanishes gradually with the decrease of layer width (i.e., increase of the total number of layers), and eventually a rather homogeneous microstructure with keyhole pores was obtained. It is suggested that the HE region has a wider influence area than the pre-set layer width.
3. Multiple A↔M transformation peaks covering a wide temperature range were observed in the layer-structured NiTi alloys, since different layers give different phase transformation behaviors. The transformation peaks gradually merge with the decrease of layer width, and the samples with layer width <300 µm show only one A↔M peak.
4. The functionally graded NiTi alloys show high damping performance (tan δ > 0.04 at oscillation frequency of 1 Hz and strain amplitude of 3 × 10^−3^) over a notably wider temperature range (up to 70 K) than homogeneous L-PBF samples, due to the continuous martensite transformation.
5. Maximizing the high-damping temperature window requires optimization of the fabrication process, since the width of the window decreases gradually with the decrease of layer width due to the homogenization of the microstructure.

The obtained results show that laser powder bed fusion is an efficient method of fabricating functionally graded NiTi alloys with a wide phase transformation interval. Good damping properties over a wide temperature range could thus be obtained. The wide working temperature window, together with the ability of fabricating complex structures is expected to promote large applications of NiTi alloys. This work may provide new insights to fabricated functionally graded materials or composite structures. However, the mechanical/functional properties of the functionally graded materials, which are essential for practical applications, have not been studied in this work. Future work is required to study the shape memory effect or superelasticity of the functionally graded NiTi alloys.

## Figures and Tables

**Table 1:** Chemical composition of NiTi alloy powders *.

Samples	Ni (wt.%)	Ni (at.%)	Ti (wt.%)	Fe (wt.%)	C (wt.%)	O (wt.%)	N (wt.%)
Powders	55.64	50.6	Bal.	< 0.005	0.0025	0.05	0.003

* The chemical composition is given by the supplier.

**Table 2:** The L-PBF process parameters for fabricating the functionally graded NiTi samples ^1^.

Samples	Scanning Speed (mm/s) ^2^	Number of Layers	Layer Width (µm) ^3^
V1	400	1	3000
V2	600	1	3000
V3	1000	1	3000
2S–2L	400 (HE), 1000 (LE)	2	1500
2S–4L	400 (HE), 1000 (LE)	4	750
2S–6L	400 (HE), 1000 (LE)	6	500
2S–8L	400 (HE), 1000 (LE)	8	375
2S–10L	400 (HE), 1000 (LE)	10	300
2S–12L	400 (HE), 1000 (LE)	12	250
2S–14L	400 (HE), 1000 (LE)	14	215
3S–3L	400 (HE), 600 (ME), 1000 (LE)	3	1000
3S–6L	400 (HE), 600 (ME), 1000 (LE)	6	500
3S–10L	400 (HE), 600 (ME), 1000 (LE)	10	300
3S–15L	400 (HE), 600 (ME), 1000 (LE)	15	200

^1^ The laser power of 120 W and hatch spacing of 80 µm are fixed. ^2^ HE, ME, and LE represent the layers manufactured with *v* = 400, 600, and 1000 mm/s, respectively. ^3^ The width of each layer is pre-set in the digital model of each sample.

## Data Availability

Not applicable.

## Supplementary Materials

The following supporting information can be downloaded at: https://www.mdpi.com/article/10.3390/ma15145073/s1. Figure S1: Partial DSC tests of the layer-structured NiTi alloy fabricated by alternating two sets of L-PBF process parameters. The sample was first cooled from 423 K to 273 K, leading to the appearance of Peak 1. Then the sample was heated up from 273 K to 423 K, leading to the appearance of Peak 2. Afterwards, the sample was cooled again to 123 K, followed by heated up to 423 K. It shows that Peak 1 and Peak 2 are a pair, and Peak 3 and Peak 4 are a pair, and they both relate to the B2-B19′ transformation, due to the large thermal hysteresis. Figure S2: The internal friction (tan δ) during heating process of: (a) the L-PBF fabricated NiTi samples with *v* = 400 (sample V1) and 1000 mm/s (sample V3), and the layer-structured samples fabricated using 2 sets of L-PBF parameters (*v* = 400, 1000 mm/s) with different total number of layers; (b) the L-PBF fabricated NiTi samples with *v* = 400 (sample V1), 600 (sample V2) and 1000 mm/s (sample V3), and the layer-structured samples fabricated using 3 sets of L-PBF parameters (*v* = 400, 600, and 1000 mm/s) with different total number of layers. Figure S3: The storage modulus measured during cooling (a) and heating (c) of the L-PBF fabricated NiTi samples with *v* = 400 (sample V1) and 1000 mm/s (sample V3), and the layer-structured samples fabricated using 2 sets of L-PBF parameters (*v* = 400, 1000 mm/s) with different total number of layers. The storage modulus measured during cooling (b) and heating (d) of the L-PBF fabricated NiTi samples with *v* = 400 (sample V1), 600 (sample V2) and 1000 mm/s (sample V3), and the layer-structured samples fabricated using 3 sets of L-PBF parameters (*v* = 400, 600, and 1000 mm/s) with different total number of layers.
