# Supplementary material for: Structure, Martensitic Transformation, and Damping Properties of Functionally Graded NiTi Shape Memory Alloys Fabricated by Laser Powder Bed Fusion
Source: Materials (Basel). 2022 Jul 21;15(14):5073. doi: 10.3390/ma15145073 (PMC9319330; doi:10.3390/ma15145073)
Supplement: Supplementary file 1 [file materials-15-05073-s001.zip › materials-1770789-supplementary.pdf]

Article

Structure, martensitic transformation, and damping properties of functionally graded NiTi shape memory alloys fabricated by laser powder bed fusion

Hao Jiang [1], Rui Xi [1], Xiaoqiang Li [1], Sergey Kustov [2], Jan Van Humbeeck [3] and Xiebin Wang [1,*]

[1] Key Laboratory for Liquid-Solid Structural Evolution and Processing of Materials (Ministry of Education), Shandong University, Jingshi Road 17923, Jinan 250061, China; Hao.Jiang2020@hotmail.com (H. Jiang); Rui.Xi2020@hotmail.com (R. Xi); lixiaoqiang3314@163.com (X. Li)
[2] Departament de Física, Universitat de les Illes Balears, Cra Valldemossa km 7.5, Palma de Mallorca E07122, Spain; Sergey.Kustov@uib.es (S. Kustov)
[3] Department of Materials Engineering, University of Leuven (KU Leuven), Kasteelpark Arenberg 44 bus 2450, Heverlee B3001, Belgium; Jan.Vanhumbeeck@kuleuven.be (J. Van Humbeeck)
* Correspondence: Xiebin.Wang@email.sdu.edu.cn; Tel.: +86 531 883 92621

1. Partial DSC tests of the layer-structured functionally graded NiTi alloys

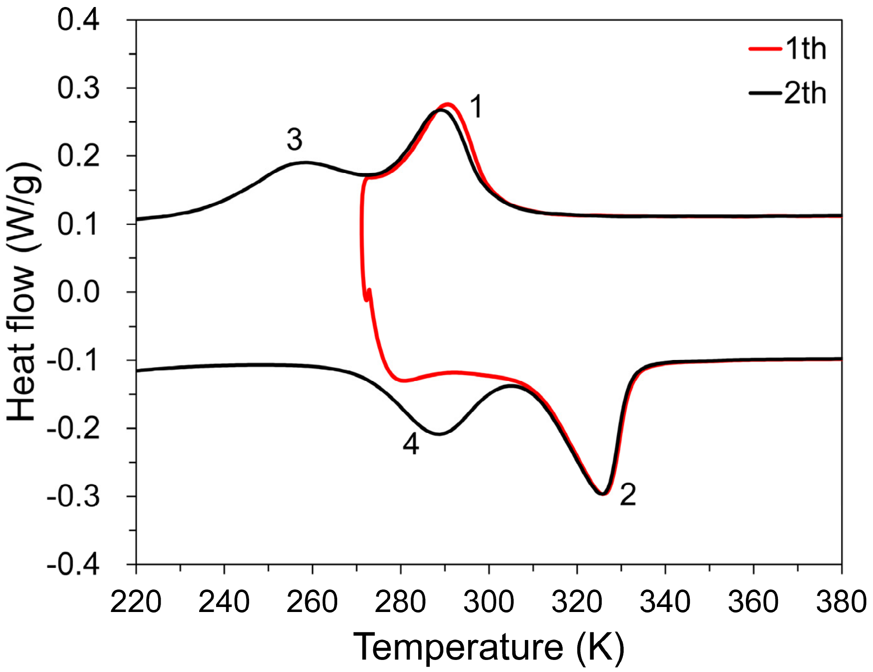

Figure S1. Partial DSC tests of the layer-structured NiTi alloy fabricated by alternating two sets of L-PBF process parameters. The sample was first cooled from 423 K to 273 K, leading to the appearance of Peak 1. Then the sample was heated up from 273 K to 423 K, leading to the appearance of Peak 2. Afterwards, the sample was cooled again to 123 K, followed by heated up to 423 K. It shows that Peak 1 and Peak 2 are a pair, and Peak 3 and Peak 4 are a pair, and they both relate to the B2-B19′ transformation, due to the large thermal hysteresis.

2. Damping properties of the layer-structured functionally graded NiTi alloys during heating

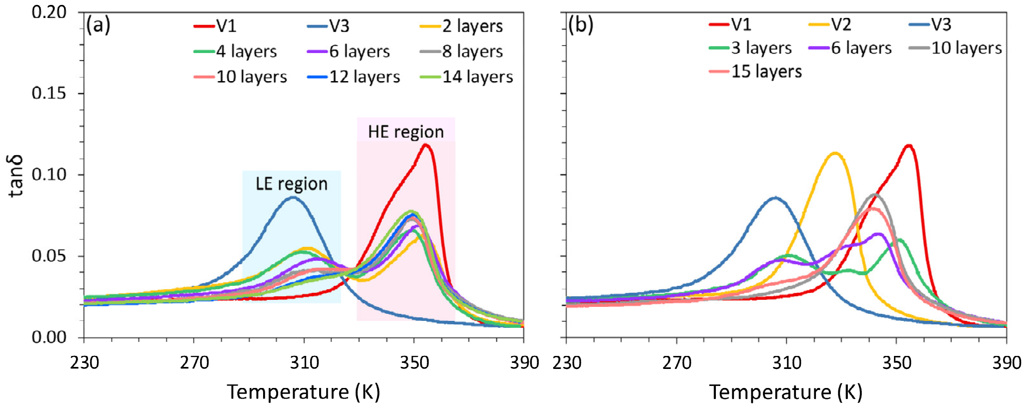

Figure S2. The internal friction (tan δ) during heating process of: (a) the L-PBF fabricated NiTi samples with v = 400 (sample V1) and 1000 mm/s (sample V3), and the layer-structured samples fabricated using 2 sets of L-PBF parameters (v = 400, 1000 mm/s) with different total number of layers; (b) the L-PBF fabricated NiTi samples with v = 400 (sample V1), 600 (sample V2) and 1000 mm/s (sample V3), and the layer-structured samples fabricated using 3 sets of L-PBF parameters (v = 400, 600, and 1000 mm/s) with different total number of layers.

3. Storage modulus of the layer-structured NiTi alloys during cooling and heating

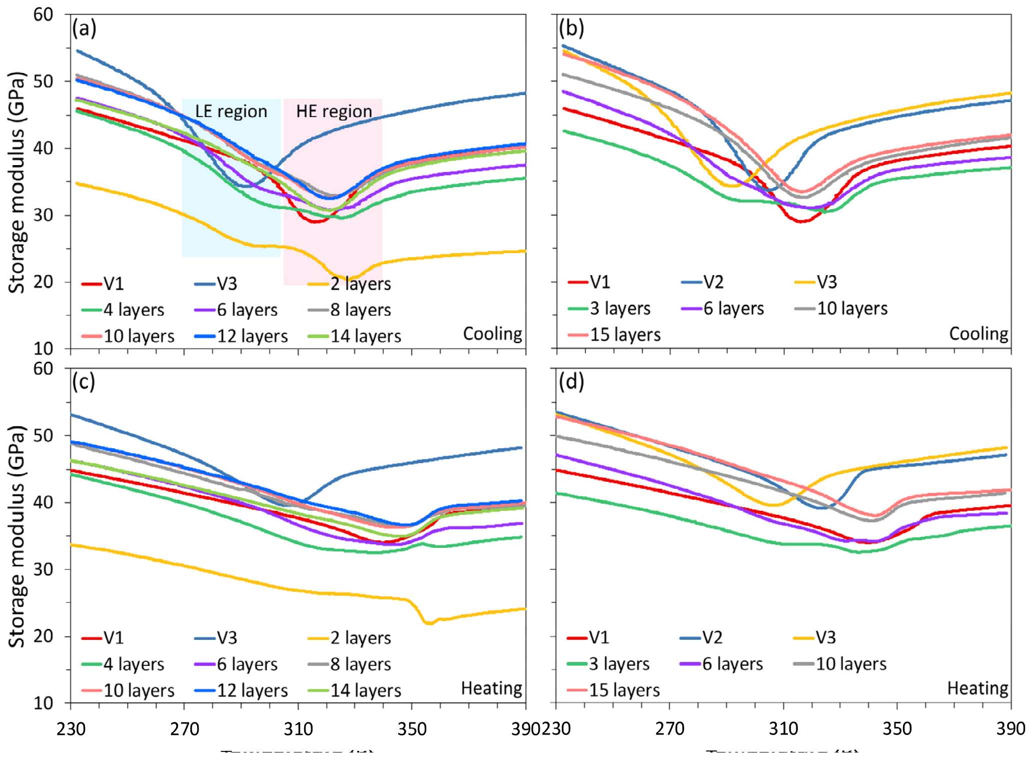

Figure S3. The storage modulus measured during cooling (a) and heating (c) of the L-PBF fabricated NiTi samples with v = 400 (sample V1) and 1000 mm/s (sample V3), and the layer-structured samples fabricated using 2 sets of L-PBF parameters (v = 400, 1000 mm/s) with different total number of layers. The storage modulus measured during cooling (b) and heating (d) of the L-PBF fabricated NiTi samples with v = 400 (sample V1), 600 (sample V2) and 1000 mm/s (sample V3), and the layer-structured samples fabricated using 3 sets of L-PBF parameters (v = 400, 600, and 1000 mm/s) with different total number of layers.
